# Mucilage protects the planktonic desmid *Staurodesmus* sp. against parasite attack by a chytrid fungus

**DOI:** 10.1093/plankt/fbac071

**Published:** 2022-12-27

**Authors:** Silke Van Den Wyngaert, Martin J Kainz, Robert Ptacnik

**Affiliations:** Wassercluster – Biologische Station Lunz, Dr Carl Kupelwieser Promenade 5, 3293 Lunz Am See, Austria; Department of Biology, University of Turku, Vesilinnantie 5, 20014 Turku, Finland; Wassercluster – Biologische Station Lunz, Dr Carl Kupelwieser Promenade 5, 3293 Lunz Am See, Austria; Department of Biomedical Research, Danube University, Dr Karl Dorrek Strasse 20, 3500 Krems, Austria; Wassercluster – Biologische Station Lunz, Dr Carl Kupelwieser Promenade 5, 3293 Lunz Am See, Austria

**Keywords:** *Staurastrum*, *Staurodesmus*, chytrids, fungal parasites, mucilage

## Abstract

Zoosporic fungi of the phylum Chytridiomycota are ubiquitous parasites of phytoplankton in aquatic ecosystems, but little is known about phytoplankton defense strategies against parasitic chytrid attacks. Using a model chytrid-phytoplankton pathosystem, we experimentally tested the hypothesis that the mucilage envelope of a mucilage-forming desmid species provides protection against the parasitic chytrid *Staurastromyces oculus*. Mucilage-forming *Staurodesmu*s cells were not accessible to the chytrid, whereas physical removal of the mucilage envelope rendered the same *Staurodesmus* sp. strain equally susceptible to chytrid infections as the original non-mucilage-forming host *Staurastrum* sp. Epidemic spread of the parasite only occurred in *Staurastrum* sp., whereas non-mucilage-bearing *Staurodesmus* sp. allowed for co-existence of host and parasite, and mucilage-bearing *Staurodesmus* sp. caused parasite extinction. In addition to the mucilage defense barrier, we also demonstrate the ability of both *Staurastrum* sp. and *Staurodesmus* sp. to resist infection by preventing chytrid development while still remaining viable and being able to reproduce and thus recover from an infection. This study extends our knowledge on phytoplankton defense traits and the functional role of mucilage in phytoplankton as a physical barrier against fungal parasites.

## INTRODUCTION

Parasitism is one of the most common consumer strategy among organisms (Lafferty *et al*., 2008) and represents an important biotic driver for host populations. Phytoplankton are key for the global carbon cycle, contributing about half of the global primary productivity (Falkowski, 1994), and are susceptible to various parasites (Reynolds, 2006). Zoosporic fungal parasites of the phylum Chytridiomycota (hereafter chytrids) are ubiquitous and highly diverse in aquatic ecosystems, infecting all major phytoplankton taxa (Frenken *et al*., 2017; Van den Wyngaert *et al*., 2022). The general life cycle of chytrid parasites involves free-living motile zoospores that serve as primary mechanisms for dispersal and transmission. Successful host localization and recognition leads to zoospore attachment and encystment on the host cell surface. The parasite penetrates into the host’s interior via rhizoids and uses host-derived nutrients to produce new zoospores that are released into the ambient water upon maturation. Non-favorable conditions can induce asexual or sexual production of metabolic inactive resting spores (Van den Wyngaert *et al*., 2017). The recent integration of chytrids in the Plankton Ecology Group model exemplifies the emerging recognition of chytrids as ecological and evolutionary drivers of phytoplankton bloom dynamics (Rasconi *et al*., 2012; Sommer *et al*., 2012). Chytrid outbreaks can cause premature termination or suppression of phytoplankton blooms (Canter and Lund, 1951; Ibelings *et al*., 2011; Gsell *et al*., 2013a). Selective chytrid parasitism can alter intra- and interspecific competition, affecting phytoplankton diversity, coexistence and succession (Canter and Lund, 1951, van Donk and Ringelberg, 1983, Gsell *et al*., 2013b). Furthermore, chytrids modify microbial interactions and the fate of photosynthetic carbon by bypassing the microbial loop and enhancing herbivory (Gerphagnon *et al*., 2019, Rasconi *et al*., 2020) via the fungal shunt (Klawonn *et al*., 2021) and the mycoloop (Kagami *et al*., 2007).

Despite this general appraisal, there is very little understanding about host specificity in phytoplankton-chytrid interactions and how phytoplankton can defend themselves against chytrid attacks. Host defense can generally be classified in three main groups; (i) behavioral defense where hosts avoid being encountered or recognized by their parasites (Parker *et al*., 2011), (ii) barrier defense that protects the host against the entry of the parasite into the cell (Underwood, 2012) and (iii) immune defense which is crucial once parasites have overcome physical host barriers (Jones and Dangl, 2006). Within populations of the diatom *Asterionella formosa*, some host cells have been shown to mount a hypersensitive death reaction in response to chytrid infection and thereby also kill the chytrid parasite before it can complete its life cycle (Canter and Jaworski, 1979), whereas toxins produced by the cyanobacterium *Planktothrix rubescens* can decrease the susceptibility of *P. rubescens* to chytrid infection (Rohrlack *et al*., 2013). By regulating buoyancy, *P. rubescens* can migrate to the metalimnion of clear water lakes, where low temperatures and light provide an environmental refuge against the chytrid parasite *Rhizophydium megarrhizum* (Rohrlack, 2018).

Desmids are a highly diverse group of unicellular green microalgae with estimates ranging from 3000 to 6000 species worldwide (Coesel and Krienitz, 2008). Species of the genera *Staurastrum* and *Staurodesmus* are particularly well-known for their wide occurrence in the euplankton (i.e. plankton in open water) across lakes and reservoirs from oligo-mesotrophic to eutrophic conditions (Kremer *et al*., 2016). They occasionally form dense blooms in summer to early fall (Kagami and Urabe, 2002; Znachor *et al*., 2016) during which chytrid infections have been observed on several species of *Staurastrum* and *Staurodesmus* (Canter, 1961; Canter and Lund, 1969; Kagami and Urabe, 2002). The summer stratification period is often characterized by low nutrient concentrations and high interspecific competition (Wetzel, 2001) and parasitic chytrids are likely to play a significant role in shaping the summer phytoplankton community structure (Canter and Lund, 1969).

Mucilage production is a common trait in desmids and many other phytoplankton taxa (Reynolds, 2006). Reynolds (2007) reviewed functions and benefits of mucilage layers for algae, including buoyant properties (reducing sinking speed), a reducing microenvironment as protection against oxidative stress, defense against digestion by grazers and nutrient sequestration or production in response to nutrient deficiency. Surprisingly, however, the role of mucilage to defend against parasites has so far not been addressed. Therefore, the aim of this study was to experimentally investigate the role of a mucilage envelope to provide protection against parasitic chytrids. We hypothesized that the mucilage envelope of the mucilage-forming desmid species, *Staurodesmu*s sp., functions as a defensive barrier against chytrid infection.

## MATERIALS AND METHODS

### Cultures

The chytrid-phytoplankton study system was comprised of the chytrid parasite *Staurastromyces oculus* (strain STAU-CHY3), its original desmid host *Staurastrum* sp. (strain STAU1) (a non-mucilage producer) and the mucilage-producing desmid *Staurodesmus* sp. (strain Staurodesmus A). *Staurastromyces oculus and Staurastrum* sp. were both isolated from Lake Stechlin (Northern Germany) in July 2015 (Van den Wyngaert *et al*., 2017). *Staurodesmus* sp. was isolated from Lake Lunz (Austria) in November 2019.

Both desmid cultures and the *Staurastrum*-chytrid co-culture were maintained in WC medium (Guillard and Lorenzen, 1972) at 18°C. The light regime was 16:8 h, providing ~ 100 μE s^−1^ m^−2^ during the 16-h light phase. All cultures are uniclonal but non-axenic. All experiments were performed in WC medium and under the same temperature and light conditions as described above.

### Mucilage removal and cell counts

**Fig. 1:**
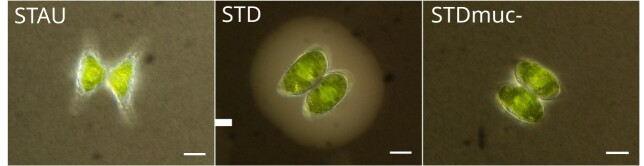
Micrographs of the different experimental host types. Indian ink staining indicates the absence of a mucilage envelope in *Staurastrum* (STAU), presence of an extensive mucilage envelope in *Staurodesmus* (STD) and the efficient removal of the mucilage envelope after sonication (STDmuc-). Scale bar = 10 μm.

To test the main hypothesis that the mucilage envelope of *Staurodesmu*s sp. provides protection against chytrid infection, the mucilage envelope was mechanically removed from *Staurodesmus* cells by ultrasonication with a Branson S250D Sonifier (Branson Ultrasonics). Sonication efficiency was evaluated in a series of preliminary experiments by staining cell suspensions with Indian ink (John *et al*., 2002) to visualize mucilage envelopes and subsequent observation under a microscope (LSM710, Zeiss, Germany) using transmitted light, prior and after the sonication treatment (Fig. 1). A sonication protocol of 1 min duration, 30% amplitude with 1 s pulse resulted in optimal removal of the mucilage envelop (Fig. 1) without causing cell damage (as cell growth continued after sonication, see Supplementary Fig. S1) and was used in all experimental set-ups.

Samples for enumeration of cell abundances and infection parameters were fixed with neutral Lugol’s solution (10 μL mL^−1^) and stored in cool and dark until further processing. For each sample, a minimum of 350 host cells were counted at ×400 magnification using an inverted microscope (DMI3000 B, Leica Microsystems, Wetzlar, Germany) according to the Utermöhl method (Lund *et al*., 1958). Chlorophyll fluorescence (Ft) as proxy for chlorophyll-a concentrations in the cultures, and photosynthetic quantum yield (Fv/Fm) of dark adapted samples (15 min) were measured with a Pulse-Amplitude-Modulation (PAM) fluorometer (AquaPen-C-AP-C100).

### Experiment 1 set-up: host susceptibility and parasite infection success

A short-term (24 h) infection experiment was performed to compare susceptibility and parasite infection success on populations of *Staurodesmus* with a mucilage envelope (STD), *Staurodesmus* sp. without a mucilage envelope (STDmuc-) and the original host *Staurastrum* sp. (STAU).

STAU and STD were pre-grown in sterile, polystyrene cell culture flasks (Greiner Bio-one) in a volume of 300 mL and were collected during exponential growth phase. STD was subdivided in three batches of 50 mL each: one batch was left untreated (STD), the second batch was sonicated (STDmuc-) and the third batch was sonicated and cells were afterwards centrifuged (18°C, 800 rcf, 15 min) and washed twice using WC medium (STDmuc-cw). The rationale behind this additional treatment was to remove residual substances of the mucilage, which could potentially interfere with parasite infectivity and obscure the direct effect of the mucilage envelope around cells. The supernatant containing the mucilage substances was collected and stored at 4°C for Experiment 2.

Experimental batches (5000 cells mL^−1^) were prepared by dilution with WC medium. Photosynthetic quantum yield (Fv/Fm) was measured after dark adaptation to verify the physiological state of the experimental populations at the start before inoculation and at the end of the experiment (see Supplementary Table S1). A parasite zoospore suspension was obtained by filtering a 7-day-old *Staurastrum*-chytrid co-culture over a plankton net (10-μm mesh-size). Zoospore survival and swimming activity were confirmed by microscopic inspection of the suspension. The experiment was conducted in 12-well plates with wells containing 3 mL of host cell suspension (final density 3750 cells mL^−1^) and 1 mL of zoospore suspension (final density 5000 zsp mL^−1^). This resulted in a 1.3:1 ratio of zoospores:host cells and ensured that parasites were not limited by host availability. The four experimental host populations (STAU, STD, STDmuc-, STDmuc-cw) exposed to parasite zoospores were replicated four times resulting in a total of 16 experimental units. Non-parasite-exposed host populations served as a control.

Each sample was counted for abundance of; (a) living uninfected host cells mL (uninf:live); (b) dead uninfected host cells mL^−1^ (uninf:dead); (c) infected host cells mL^−1^ carrying one or more infection(s) (inf); (d) number of attached zoospores mL^−1^ (zsp); and (e) infected host cells with resistance phenotype mL^−1^ (inf:resist). We categorized uninf:live cells as those cells without an attached chytrid and intact, green chloroplasts, whereas uninf:dead cells were categorized as empty host cells deprived of most cellular content without an attached chytrid. The resistance phenotype was characterized by the host cell bearing an attached, but undeveloped chytrid spore similar in size as a zoospore but deprived from any cellular content, and an orange-brown pigmented structure within the host cell at the spot of parasite penetration surrounded by a variable extended, hyaline barrier zone (Sparrow, 1960; Cook, 1963) (see Fig. 4A). The proportion of infected cells with a resistance phenotype was calculated as inf:resist/(inf + inf:resist). Parasite infection success, i.e. proportion of attached zoospores, was calculated as the abundance of attached zoospores (zsp) divided by the initially added zoospore abundance. Infection prevalence, i.e. proportion of cells carrying live infections (excluding host cells with resistance phenotype) was calculated as inf/(uninf:live+inf). The intensity of infection was defined as the average number of attached zoospores per infected host cell. To analyze if parasite infections followed a random, uniform or aggregated distribution, the number of host cells with *n* attached fungi (*n* = 0, 1, 2, … ) was counted according to Yoneya *et al*. (2021).

### Experiment 2 set-up: effect of mucilage residues on parasite development

To evaluate if exposure to the mucilage residues of *Staurodesmus* sp. affects parasite infectivity, the original STAU host population was incubated using the supernatant from STD after sonication and centrifugation (see STDmuc-cw treatment in Experiment 1). The presence of mucilage substances in the supernatant was verified by Indian ink staining and light microscopy. The experiment was conducted in 12-well plates containing 2.75 mL of mucilage supernatant or WC medium, 0.25 mL STAU (final density 4000 cells mL^−1^) and 1 mL zoospore suspension (obtained as described in Experiment 1). In addition, non-parasite-exposed STAU served as control for the effect of STD mucilage residues on STAU growth performance.

After 7 days of incubation, a sample (2 mL) was taken for measurements of chlorophyll fluorescence (Ft) as proxy for abundance of chlorophyll containing cells, and photosynthetic quantum yield (Fv/Fm). Another sample (2 mL) was fixed (Lugol) for evaluating the prevalence of infection.

### Experiment 3 set-up: parasite development and infection dynamic

A 7-day infection experiment was performed to compare the ability of the chytrid to develop and transmit within STD, STDmuc- populations and the original host population STAU. Experimental host populations were prepared as described in Experiment 1, except that the STDmuc-cw treatment was omitted because Exps 1 and 2 showed no evidence that mucilage residues negatively affected parasite infectivity or STAU growth (see Results). Because we observed fast mucilage regeneration in *Staurodesmus* (i.e. original thickness of mucilage envelope was regenerated within 24 h, see Supplementary Fig. S2), we included a treatment in which 10% of the volume of STDmuc- was replaced daily with freshly sonicated *Staurodesmus* cells at same densities (based on Chlorophyll fluorescence measurement). To account for the effect of concomitant parasite removal, we performed this daily replacement also in STAU and STD chytrid-exposed treatments.

Fifty ml cell culture flasks were initially filled with 45 mL host cell suspensions (1500 cells mL^−1^). In the parasite-exposed treatments and non-parasite-exposed treatments, 1 mL of a 7-day-old *Staurastrum*-chytrid co-culture with nearly 100% prevalence of infection and 1 mL of WC medium were added, respectively. All treatments were replicated three times resulting in a total of 30 experimental units. Each unit was shaken manually twice a day and their position randomized. Subsamples were taken on Days 0, 3 and 7, except for samples for fluorescence and photosynthetic quantum yield, which were taken daily. Lugol fixed subsamples were counted for abundance and infection parameters as described in Experiment 1. To test viability of host cells with resistance phenotype and their ability to reproduce, 12 single cells were manually isolated from the STDmuc- population at Day 7 and transferred into individual wells of a 24-well plate filled with 1 mL of WC medium. Viability and growth were verified after 2 days and after 12 days using an inverted light microscope.

### Data analysis

One-way analysis of variance (ANOVA) was used to analyze the effect of host type (Exp 1) on infection parameters and the effect of mucilage (Exp 2) on *Staurastrum* growth. For Experiment 3, repeated measure ANOVA was conducted to analyze the effect of host type and replacement treatment on prevalence of infection. Infected host cells with resistance phenotype were only quantified at the end of the experiment (on Day 7) and was analyzed by a two-way ANOVA. Pairwise comparisons were conducted using the Tukey’s post hoc test. All variables were tested for normality and equal variance using the Shapiro–Wilk and Levene’s tests, respectively. Relative data (%) were arcsin (prevalence of infection) and log+1 (proportion resistant cells) transformed to meet assumptions of normality. To evaluate if the distribution of parasite infections in Exp 1 was random (following Poisson distribution), aggregated or uniform, we used the standardized Morisita index of aggregation (Imst), using the function dispindmorisita, available in the vegan package in R (Oksanen *et al*., 2020). When the index value Imst < −0.5, the distribution is uniform, whereas the distribution is aggregated when Imst > 0.5. When −0.5 ≤ Imst ≤ 0.5, the distribution is Poisson (Smith-Gill, 1975). The overall effect of host population on parasite aggregation was analyzed by Kruskal–Wallis test, followed by pairwise Wilcoxon rank sum tests with Benjamini–Hochberg adjusted *P* values. All statistical analyses were performed with R version 4.1.2.

## RESULTS

### Experiment 1: host susceptibility and parasite infection success

**Fig. 2:**
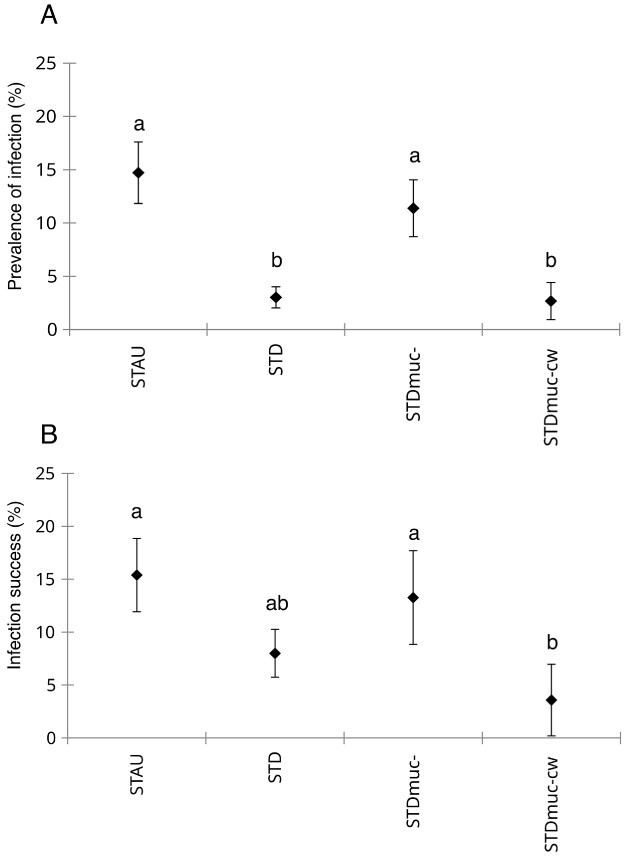
Prevalence of infection (A) and parasite infection success (B) in the four experimental host populations of Exp1. Different letters (a, b) represent significant differences, whereas similar letters (a, a) represent non-significant differences. Error bars denote mean SD (*n* = 4).

**Fig. 3:**
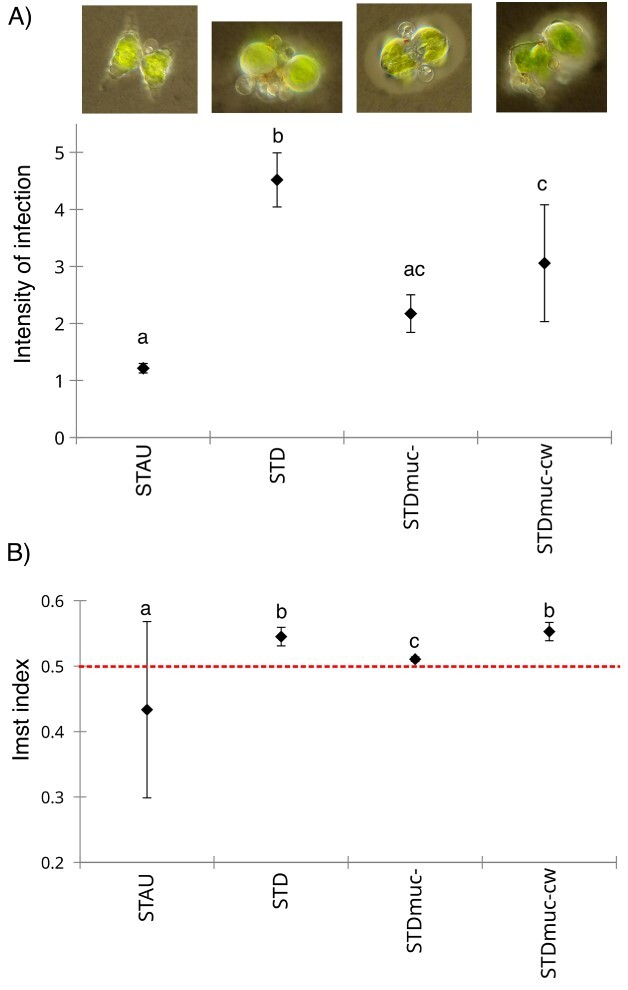
Intensity of infection (A) and standardized Morisita index of aggregation (B) for the four experimental host populations of Exp1. Values above dashed red line indicates aggregated parasite distribution, whereas values below the line indicates random parasite distribution. Different letters (a, b, c) represent significant differences, whereas similar letters (b, b) represent non-significant differences. Error bars denote mean SD (*n* = 4). Micrographs above the figure plot exemplify the infection phenotypes on the different experimental host populations, from mainly single infected *Staurastrum* (STAU) cells (i.e. low intensity of infection and a more random parasite distribution) towards increasing multiple infections in *Staurodesmus* (STD) cells (i.e. higher intensity of infection and a more aggregated parasite distribution).

Prevalence of infection (Pr%) was highest on the original host STAU (15 ± 3%), followed by STDmuc- (11 ± 3%) and lowest on STD (3 ± 1%) and STDmuc-cw (3 ± 2%). Pr% in STD and STDmuc-cw was significantly lower compared with STAU and STDmuc-, whereas Pr% did not significantly differ between STAU and STDmuc- (Table I, Fig. 2A). The parasite infection success showed the same pattern, except that there was no statistical difference between STD and STDmuc- (Table I, Fig. 2B). Intensity of infection was significantly higher in STD (4.52 ± 0.47) compared with STAU (1.21 ± 0.08), STDmuc- (2.17 ± 0.33) and STDmuc-cw (3.06 ± 1.03) (Table I, Fig. 3A). The infection distribution differed between host populations (Chi-square = 12.79, *P* = 0.005, Table I). The Imst index for three out of four STAU replicates was at (0.50) or below (0.23) the confidence limit of a Poisson distribution, whereas one replicate was slightly above the confidence limit (0.501) (see Supplementary Table S2), overall supporting a more random parasite distribution in STAU (Imst index = 0.43 ± 0.13, Fig. 3B). Chi-squared test was also non-significant (i.e. no deviation from Poisson distribution) for three out of four STAU replicates and only one replicate (1 with highest Imst index) had a non-random infection distribution, whereas deviation from a Poisson distribution (Chi-square test *P* = < 0.001) was observed in all other host populations (see Supplementary Table S2) with strongest aggregation in STD and STDmuc-cw (Imst index = 0.55 ± 0.014) and weaker aggregation in STDmuc- (Imst index = 0.51 ±  0.004) (Fig. 3B).

**Table I:** Statistical output of Experiment 1: one-way ANOVA and non-parametric Kruskal–Wallis test of host type effects on different infection variables and their respective post hoc tests

Exp1	One-way ANOVA				
Dependent variable	*F*(3,12)	*P*	Tukey HSD	95% C.I.	*P* adj
Prevalence of infection %	30.03	**<0.001**	STAU/STD	[−16.34,-7.06]	**<0.001**
			STAU/STDmuc-	[−7.96,1.31]	0.20
			STAU/STDmuc-cw	[−16.69,-7.41]	**<0.001**
			STD/STDmuc-	[3.73,13.01]	**<0.001**
			STD/STDmuc-cw	[−4.99,4.29]	0.99
			STDmuc-/STDmuc-cw	[−13.36,-4.08]	**<0.001**
Infection success %	9.41	**0.002**	STAU/STD	[−14.68,-0.11]	*0.05*
			STAU/STDmuc-	[−9.41,5.16]	0.82
			STAU/STDmuc-cw	[−19.10,-4.53]	**0.002**
			STD/STDmuc-	[−2.02,12.55]	0.19
			STD/STDmuc-cw	[−11.70,2.86]	0.32
			STDmuc-/STDmuc-cw	[−16.97,-2.40]	**0.009**
Intensity of infection	22.64	**<0.001**	STAU/STD		**<0.001**
			STAU/STDmuc-		0.15
			STAU/STDmuc-cw		**0.004**
			STD/STDmuc-		**<0.001**
			STD/STDmuc-cw		**0.02**
			STDmuc-/STDmuc-cw		0.20
Proportion resistant cells %	2.23	0.118			
	**Kruskal–Wallis**		**Pairwise Wilcoxon rank sum**		
Parasite aggregation (Imst)	Chi-square = 12.79 df = 3	**0.005**	STAU/STD	NA	**0.034**
			STAU/STDmuc-	NA	**0.034**
			STAU/STDmuc-cw	NA	**0.034**
			STD/STDmuc-	NA	**0.034**
			STD/STDmuc-cw	NA	0.69
			STDmuc-/STDmuc-cw	NA	**0.034**

Values in bold denote a significant effect (*P* < 0.05).

**Fig. 4:**
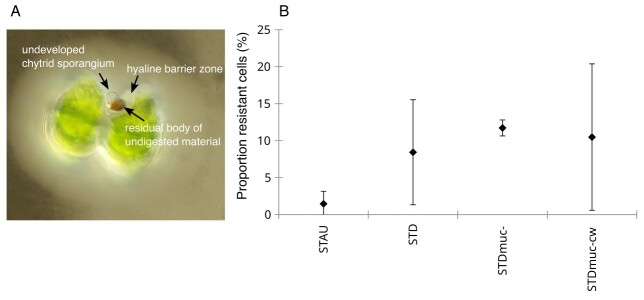
Micrograph of a Staurodesmus host cell showing a resistance phenotype (A) and the proportion of infected cells with resistance phenotype (B) in the experimental host populations of Exp1. Error bars denote mean SD (*n* = 4).

Cells with a parasite resistance phenotype were present in all of the host populations. The proportion of infected cells with resistance phenotype was non-significantly higher in the *Staurodesmus* sp. treatments (STD, STDmuc- and STDmuc-cw) as compared with STAU (Table I, Fig. 4B).

### Experiment 2: effect of mucilage residues on parasite development

After 7 days, Pr% reached 100% in both the control and mucilage-exposed STAU populations (data not shown). Non-chytrid-exposed STAU populations reached similar abundances (measured as Chlorophyll fluorescence) in both treatments (*F*(1,4) = 0.054, *P* = 0.827).

### Experiment 3: parasite infection dynamics

**Fig. 5:**
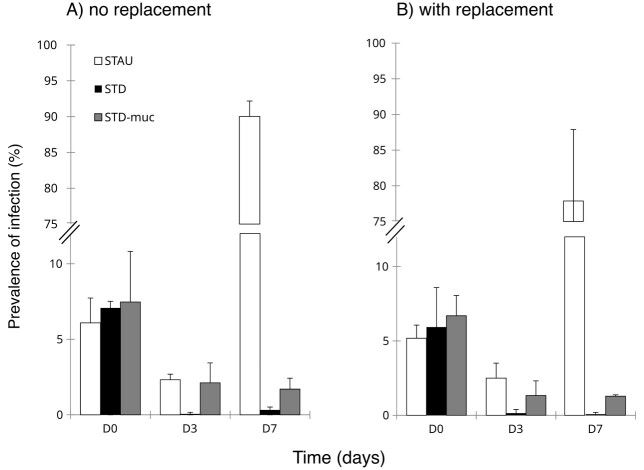
Prevalence of infection at Days 0, 3 and 7 in the “no replacement” (A) and “with replacement” (B) treatment. Error bars denote mean SD (*n* = 3).

**Fig. 6:**
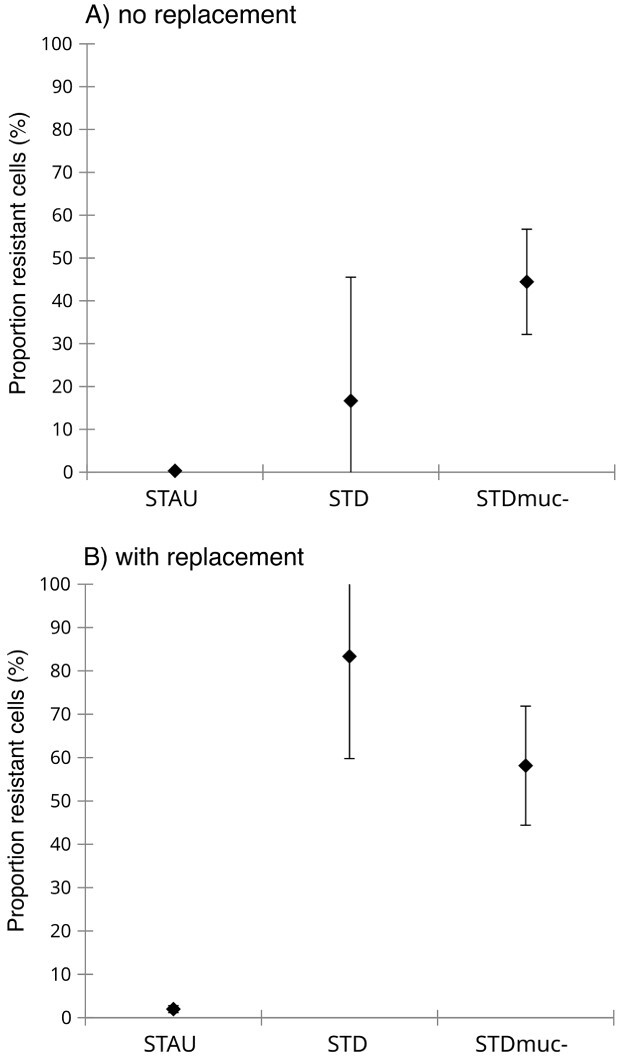
Proportion of infected cells with resistance phenotype in the “no replacement” (A) and “with replacement” (B) treatment at Day 7. Error bars denote mean SD (*n* = 3).

Starting conditions (i.e. host cell density and Pr%) were similar for all host populations and treatments [host cell density *F*(9,20) = 0.83, *P* = 0.6; infection prevalence *F*(5,12) = 0.67, *P* = 0.53, Fig. 5]. There was a significant two-way interaction between Host and Time on Pr% (Table II). The Pr% was significantly higher in STAU and STDmuc- compared with STD at Day 3, independent of the replacement treatment (Table III, Fig. 5). At the end of the experiment, a high Pr% (range: 70–92%) was only reached in STAU, whereas it remained very low in STDmuc- (range: 1–2.5%) and even lower in STD (0–0.5%). The replacement treatment resulted in a slightly lower prevalence of infection in all host populations [*F*(1,12) = 6.12, *P* = 0.03, Table II] and caused parasite extinction in two out of three replicates of STD (Fig. 5B). There were also significant main effects of host and replacement on proportion of infected cells with resistance phenotype (Table IV). The proportion of cells with a resistance phenotype at Day 7 was higher in the replacement treatment and it was significantly higher in STDmuc- compared with the original host STAU (Fig. 6, Table IV). From the 12 isolated STDmuc- cells with resistance phenotype, four showed a first cell doubling after 2 days. After 12 days, growth was observed in 8 out of 12 isolates and 4 out of 12 cells died off without having undergone cell doubling.

**Table II:** Experiment 3: repeated measures ANOVA analysis of prevalence of infection over time

source of variation	D.F.	MS	F	*P*
*Within-subjects effect*				
Time	2	0.5305	268.72	**<0.001**
Host*Time	4	0.7608	385.39	**<0.001**
Replacement *Time	2	0.0055	2.78	0.08
Host*Replacement*Time	4	0.0038	1.92	0.1397
Error	24	0.0020		
*Between-subjects effect*				
Host	2	0.9053	387.957	**<0.001**
Replacement	1	0.0142	6.105	**0.0294**
Host*Replacement	2	0.0024	1.012	0.3925
Error	12	0.0023		

Values in bold denote a significant effect (*P* < 0.05).

**Table III:** Experiment 3: effect of host type on prevalence of infection at the three different time points (Days 0, 3, 7) and post hoc tests for significant host type effects

	D.F.	MS	F	*P*	Tukey HSD: Host	95% C.I.	*P* adj
Day 0							
Host	2	0.0012	0.77	0.48	n.a.	n.a.	n.a.
Error	15	0.0015					
Day 3							
Host	2	0.0310	28.96	**<0.001**	STAU/STDmuc-	[−0.078, 0.020]	0.31
Error	15				STAU/STD	[−0.185, −0.087]	**<0.001**
					STDmuc-/STD	[0.059, 0.157]	**<0.001**
Day 7							
Host	2	2.3948	439.3	**<0.001**	STAU/STDmuc-	[−1.160, −0.938]	**<0.001**
Error	15	0.0055			STAU/STD	[−1.245, −1.024]	**<0.001**
					STDmuc-/STD	[−0.003, 0.174]	*0.06*

Values in bold denote a significant effect (*P* < 0.05).

**Table IV:** Experiment 3: two-way ANOVA analysis of the effect of host type and replacement treatment on the proportion of resistant cells at Day 7 and post hoc test for significant host type effect

	D.F	MS	F	*P*
Host	2	42.91	6.22	**0.016**
Replacement	1	56.35	8.16	**0.016**
Host*Replacement	2	23.64	3.42	0.07
Error	11	6.90		
Tukey HSD: Host	95% C.I.		*P* adj	
STAU/STDmuc-	[1.25, 9.45]		**0.012**	
STDmuc-/STD	[−1.61, 6.98]		0.25	
STAU/STD	[−1.64, 6.96]		0.26	

Values in bold denote a significant effect (*P* < 0.05).

## DISCUSSION

We provide experimental evidence that a mucilage envelope can protect *Staurodesmus* sp. against chytrid infection by acting as a physical barrier. *Staurodesmus* sp. cells surrounded by a mucilage envelope were not accessible to the parasitic chytrid *Staurastromyces oculus*, whereas the same *Staurodesmus* sp. strain without mucilage envelope became equally susceptible to chytrid infections as the original host *Staurastrum* sp. In plants, mucilage secretion by root cells (defined as the root extracellular trap) likewise provides a physical barrier against pathogens and in addition consist of various classes of anti-microbial compounds (Driouich *et al*., 2019). However, our experiment did not provide evidence that the mucilage of *Staurodesmus* sp. acted as a chemical barrier against chytrids. In fact, sonicated *Staurodesmus* cells which received additional manipulation to remove residual substances of the mucilage from the medium (STDmuc-cw) were less susceptible to infection when compared with *Staurodesmus* sp. cells that were only sonicated. Elevated physiological host stress induced by centrifugation and washing is a plausible explanation for the reduced parasite infection success observed in the STDmuc-cw treatment as other studies have shown that obligate parasitic chytrids prefer healthy, non-stressed hosts (Van den Wyngaert *et al*., 2014; Schampera *et al*., 2021).

Mucilage possession by the host cell did not affect the overall number of zoospores that attacked a host population; a similar number of zoospores were able to successfully find and attach to hosts in all four treatments in Exp 1. However, mucilage affected the distribution of parasites within the host population. Although parasites were more randomly distributed on the original host STAU (in concordance with results from Yoneya *et al*., 2021), in the STD treatment of Exp 1, they were highly aggregated on those STD cells that were (naturally) lacking a mucilage layer (see images in Fig. 3). Although the overall number of attached zoospores was not reduced in non-sonicated STD populations, parasite development on these populations was severely limited and even resulted in complete parasite extinction in the replacement treatment in Exp 3. We attribute this result to a very low proportion of susceptible hosts within STD populations leading to a highly aggregated parasite distribution which in turn lowers the parasite’s reproductive output due to a lower amount of host nutrients available per parasite (Klawonn *et al*., 2021). This in combination with parasite losses induced by the host cell replacement treatment which unavoidably diluted the parasite population, resulted in a negative parasite net growth, rendering the population to extinction (Anderson and May, 1979). The daily replacement of 10% of the *Staurodesmus* sp. population with susceptible hosts (e.g. STDmuc- cells) did not allow an epidemic spread of the chytrid; however, it prevented the chytrid from going extinct and allowed co-existence of the *Staurodesmus* sp. and parasite population.

Although our results suggest that the mucilage envelope of *Staurodesmus* sp. can provide protection against infection by the chytrid species *Staurastromyces oculus*, it may not protect against all chytrid parasites per se. Some chytrids, such as *Endocoenobium eudorinae*, *Dangeardia mamillata*, *Algomyces stechlinensis*, *Rhizophydium ubiquetum* and *Phlyctochytrium planicorne* that can grow their rhizoids through the mucilage layer of colonial green algae (Canter, 1950; Van den Wyngaert *et al*., 2018) or desmids (Canter, 1961) likely possess carbohydrate active enzymes that can degrade complex mucilage polymers (Lange *et al*., 2019). Instead of being a universal defense barrier against chytrid parasites, mucilage envelopes may thus rather reduce the number of potential parasitic chytrid species for the host. Furthermore, mucilage production is sensitive to environmental conditions and cell cycle (Surek, 1983; Reynolds, 2007). Thus, protection against chytrids in mucilage producing microalgae is likely to be context dependent.

In addition to the mucilage defense barrier, we observed the ability in both *Staurastrum* sp. and *Staurodesmus* sp. to resist infection after physical cell barriers were broken. A successful resistance response blocked chytrid development in the early infection stage and lead to parasite death. These resistant host cells showed a particular phenotype, i.e. a hyaline barrier zone surrounding a conspicuous orange-brown pigmented structure at the penetration site of the parasite which presumably prevented the chytrid to access host nutrients (Fig. 4A). Similar resistance phenotypes have been described earlier in chlorophytes and other desmid species upon attacks from chytrids and other eukaryotic parasites (Sparrow, 1960; Cook, 1963), suggesting that the defense response is widespread. However, the underlying mechanisms are currently not known. In recent years, autophagy, a highly conserved and versatile cellular degradation pathway in eukaryotes, has emerged as a key player in host–pathogen interactions during infection (Pérez-Pérez *et al*., 2012; Evans *et al*., 2018). The induction and possibly the mutual hijacking of host and pathogen autophagic processes have been shown to be central in deciding the outcome of the interaction between the oomycete pathogen *Anisolpidium ectocarpii* and the giant kelp host *Macrocystis pyrifera* (Murúa *et al*., 2020). The orange-brown pigmented material in both resistant and non-resistant (no secondary layer formation) infected host cells are most likely residual bodies of non-digested material (Seto *et al*., 2020) and indicative of (auto)phagic processes. The possible interplay of host and parasite autophagy in the outcome of desmid-chytrid interactions will need further ultrastructural and molecular investigations. Although the ability of killing the chytrid parasite during the initial infection phase by autophagic host cell death has also been observed in the diatom *A. formosa* (Canter and Jaworski, 1979), we show that *Staurodesmus* sp. can resist infection by preventing chytrid development while still remaining viable and being able to reproduce and thus recover from an infection. Interestingly, we found that the proportion of resistant host cells was higher in *Staurodesmus* sp. compared with the original host *Staurastrum* sp., which could be an additional factor explaining why the spread of infection remained very low in *Staurodesmus* despite the regular introduction of sonicated susceptible cells. This result may reflect differences in coevolutionary history and local adaptation of *Staurastromyces oculus* making it better at evading host recognition and immunity in its sympatric host *Staurastrum* sp. (Lively, 1989). Reciprocal infection experiments with sympatric and allopatric *Staurodesmus* sp.-chytrid and/or *Staurastrum* sp.-chytrid populations could test this hypothesis.

## CONCLUSION

This study furthers our understanding about the functional role of mucilage in phytoplankton. We provide experimental evidence that a mucilage envelope can protect *Staurodesmus* sp. against chytrid infection by acting as a physical barrier. In addition to the mucilage defense barrier, we demonstrate the ability of both *Staurastrum* sp. and *Staurodesmus* sp. to resist infection. To our knowledge, this is the first study showing that a phytoplankton host can resist to its chytrid parasite while still remaining viable and being able to reproduce and thus recover from an infection. Ultrastructural and molecular investigations are needed to further characterize the mechanisms involved.

## Supplementary Material

VandenWyngaert_etal_JPR_SI_revised_fbac071

## Data Availability

All data are included in this article and its supplementary information file.
