# Supplementary material for: Mucilage protects the planktonic desmid Staurodesmus sp. against parasite attack by a chytrid fungus
Source: J Plankton Res. 2022 Dec 27;45(1):3–14. doi: 10.1093/plankt/fbac071 (PMC9896892; doi:10.1093/plankt/fbac071)
Supplement: VandenWyngaert_etal_JPR_SI_revised_fbac071 [file vandenwyngaert_etal_jpr_si_revised_fbac071.zip › VandenWyngaert_etal_JPR_SI_revised_fbac071.docx]

**Supporting Information:** Mucilage protects the planktonic desmid *Staurodesmus* sp. against parasite attack by a chytrid fungus

Silke Van den Wyngaert, Martin J. Kainz, Robert Ptacnik


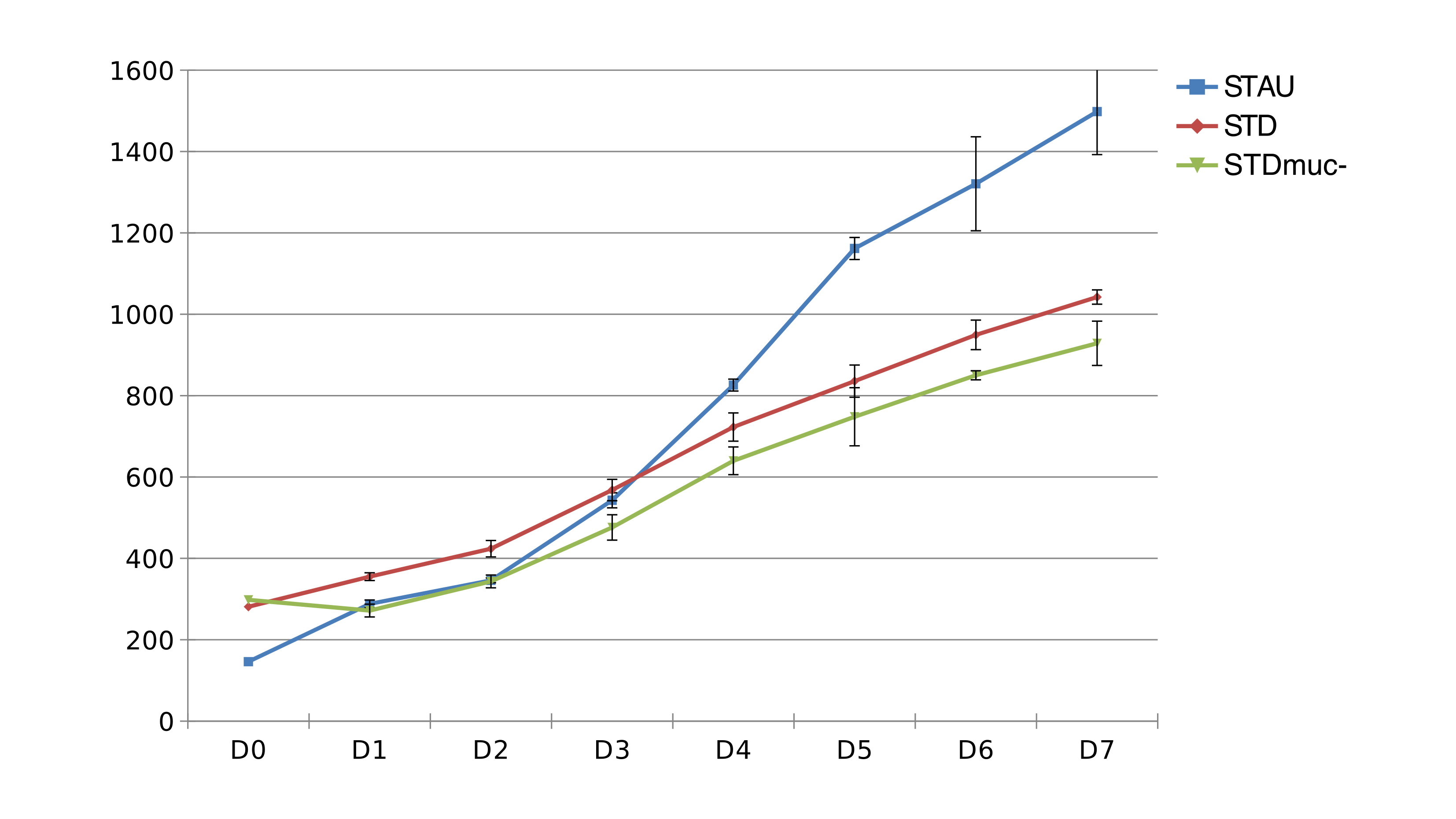
**Fig. S1** Growth curves of uninfected Staurastrum (STAU), Staurodesmus (STD) and Staurodesmus sonicated (STDmuc-), based on instantaneous chlorophyll fluorescence (Ft)


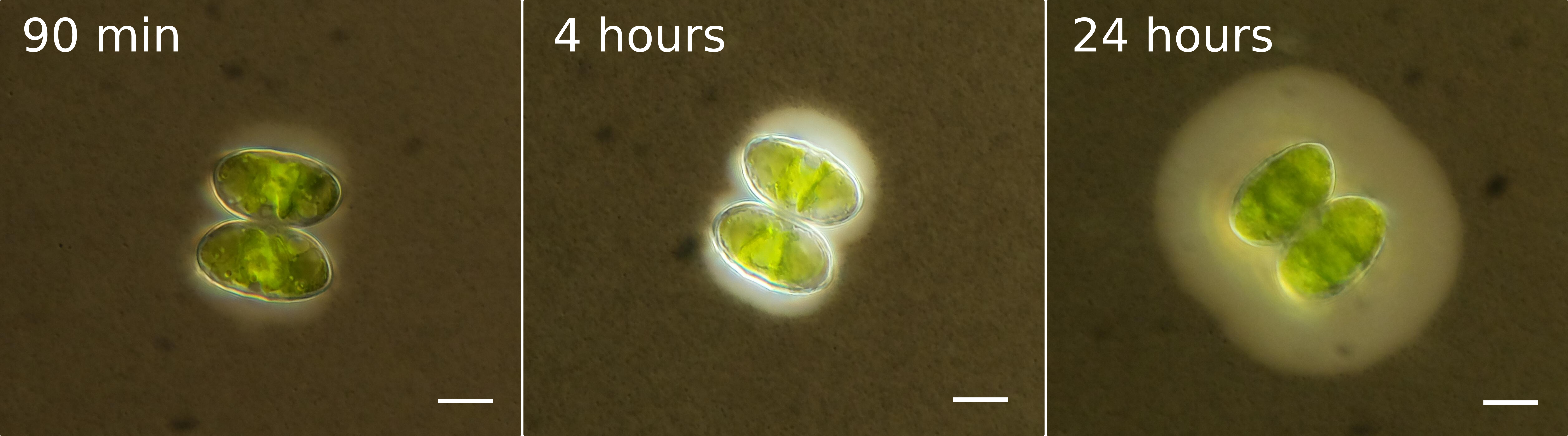


**Fig. S2** Mucilage regeneration in Staurodesmus sp.; 90min, 4h and 24h after sonication.

Scale bar = 10µm

**Table S1**: Experiment 1: Photosynthetic quantum yield (Fv/Fm)

| **Experimental host populations before experimental inoculation** |  | Fv/Fm |
| --- | --- | --- |
| STAU |  | 0.77 |
| STD |  | 0.77 |
| STD-muc |  | 0.74 |
| STD-muc-cw |  | 0.74 |
|  |  |  |
| **Experimental host populations at the end of the experiment (24hours)** | replicate |  |
| STAU | r1 | 0.77 |
| STAU | r2 | 0.74 |
| STAU | r3 | 0.75 |
| STAU | r4 | 0.73 |
| STAU+CHY | r1 | 0.73 |
| STAU+CHY | r2 | 0.73 |
| STAU+CHY | r3 | 0.72 |
| STAU+CHY | r4 | 0.73 |
| STD | r1 | 0.75 |
| STD | r2 | 0.76 |
| STD | r3 | 0.76 |
| STD | r4 | 0.75 |
| STD+CHY | r1 | 0.76 |
| STD+CHY | r2 | 0.76 |
| STD+CHY | r3 | 0.75 |
| STD+CHY | r4 | 0.76 |
| STD-muc | r1 | 0.74 |
| STD-muc | r2 | 0.74 |
| STD-muc | r3 | 0.75 |
| STD-muc | r4 | 0.75 |
| STD-muc+CHY | r1 | 0.73 |
| STD-muc+CHY | r2 | 0.73 |
| STD-muc+CHY | r3 | 0.74 |
| STD-muc+CHY | r4 | 0.74 |
| STD-muc-cw | r1 | 0.74 |
| STD-muc-cw | r2 | 0.75 |
| STD-muc-cw | r3 | 0.74 |
| STD-muc-cw | r4 | 0.75 |
| STD-muc-cw+CHY | r1 | 0.75 |
| STD-muc-cw+CHY | r2 | 0.74 |
| STD-muc-cw+CHY | r3 | 0.74 |
| STD-muc-cw+CHY | r4 | 0.75 |

**Table S2**: Experiment 1: Evaluation of parasite distribution on the different experimental host populations by the Standardized Morisita index (Imst) and chi-squared test (P_chisq_Poissoin). When −0.5 ≤ Imst ≤ 0.5 (representing the confidence limits around random distribution), the distribution does not deviate from Poisson and is thus considered random. When the p-value of the chi-square test is small (p < 0.01), the null hypothesis, i.e. that multiple infections occur at random and the infection number follows a Poisson distribution, is rejected.

| Host type | replicate | standardized morisita index (Imst) | P_chisq_Poisson |
| --- | --- | --- | --- |
| STAU | 1 | 0.500 | 0.88867 |
| STAU | 2 | 0.501 | **0.0001** |
| STAU | 3 | 0.500 | 0.78281 |
| STAU | 4 | 0.231 | 0.99993 |
| STD | 1 | 0.540 | **< 0.0001** |
| STD | 2 | 0.531 | **< 0.0001** |
| STD | 3 | 0.545 | **< 0.0001** |
| STD | 4 | 0.565 | **< 0.0001** |
| STDmuc- | 1 | 0.506 | **< 0.0001** |
| STDmuc- | 2 | 0.511 | **< 0.0001** |
| STDmuc- | 3 | 0.511 | **< 0.0001** |
| STDmuc- | 4 | 0.515 | **< 0.0001** |
| STDmuccw | 1 | 0.544 | **< 0.0001** |
| STDmuccw | 2 | 0.558 | **< 0.0001** |
| STDmuccw | 3 | 0.570 | **< 0.0001** |
| STDmuccw | 4 | 0.539 | **< 0.0001** |
